# Metabolic signatures of insulin resistance in non-diabetic individuals

**DOI:** 10.1186/s12902-022-01130-3

**Published:** 2022-08-24

**Authors:** Babak Arjmand, Saeed Ebrahimi Fana, Erfan Ghasemi, Ameneh Kazemi, Robabeh Ghodssi-Ghassemabadi, Hojat Dehghanbanadaki, Niloufar Najjar, Ardeshir Kakaii, Katayoon Forouzanfar, Ensieh Nasli-Esfahani, Farshad Farzadfar, Bagher Larijani, Farideh Razi

**Affiliations:** 1 Cell Therapy and Regenerative Medicine Research Center, Endocrinology and Metabolism Molecular-Cellular Sciences Institute, Tehran, Iran; 2 Diabetes Research Center, Endocrinology and Metabolism Clinical Sciences Institute, Tehran University of Medical Sciences, Tehran, Iran; 3 Department of Clinical Biochemistry, School of Medicine, Tehran University of Medical Sciences, Tehran, Iran; 4 Non-Communicable Diseases Research Center, Endocrinology and Metabolism Population Sciences Institute, Tehran University of Medical Sciences, Tehran, Iran; 5 Department of Biostatistics, School of Medical Sciences, Tarbiat Modares University, Tehran, Iran; 6 Metabolomics and Genomics Research Center, Endocrinology and Metabolism Molecular-Cellular Sciences Institute, Tehran University of Medical Sciences, Tehran, Iran; 7 Metabolic Disorders Research Center, Endocrinology and Metabolism Molecular -Cellular Sciences Institute, Tehran University of Medical Sciences, Tehran, Iran; 8 Endocrinology and Metabolism Research Center, Endocrinology and Metabolism Clinical Sciences Institute, Tehran University of Medical Sciences, Tehran, Iran

**Keywords:** Metabolomics, Plasma metabolite, Amino acids, Acylcarnitine, Insulin sensitivity, Insulin resistance, HOMA-IR

## Abstract

**Background:**

Insulin resistance (IR) evolved from excessive energy intake and poor energy expenditure, affecting the patient's quality of life. Amino acid and acylcarnitine metabolomic profiles have identified consistent patterns associated with metabolic disease and insulin sensitivity. Here, we have measured a wide array of metabolites (30 acylcarnitines and 20 amino acids) with the MS/MS and investigated the association of metabolic profile with insulin resistance.

**Methods:**

The study population (*n* = 403) was randomly chosen from non-diabetic participants of the Surveillance of Risk Factors of NCDs in Iran Study (STEPS 2016). STEPS 2016 is a population-based cross-sectional study conducted periodically on adults aged 18–75 years in 30 provinces of Iran. Participants were divided into two groups according to the optimal cut-off point determined by the Youden index of HOMA-IR for the diagnosis of metabolic syndrome. Associations were investigated using regression models adjusted for age, sex, and body mass index (BMI).

**Results:**

People with high IR were significantly younger, and had higher education level, BMI, waist circumference, FPG, HbA1c, ALT, triglyceride, cholesterol, non-HDL cholesterol, uric acid, and a lower HDL-C level. We observed a strong positive association of serum BCAA (valine and leucine), AAA (tyrosine, tryptophan, and phenylalanine), alanine, and C0 (free carnitine) with IR (HOMA-IR); while C18:1 (oleoyl L-carnitine) was inversely correlated with IR.

**Conclusions:**

In the present study, we identified specific metabolites linked to HOMA-IR that improved IR prediction. In summary, our study adds more evidence that a particular metabolomic profile perturbation is associated with metabolic disease and reemphasizes the significance of understanding the biochemistry and physiology which lead to these associations.

**Supplementary Information:**

The online version contains supplementary material available at 10.1186/s12902-022-01130-3.

## Introduction

Insulin resistance (IR) is a complex metabolic disorder characterized by the attenuated responsiveness of peripheral tissues, primarily muscle, liver, and adipose tissue, to insulin signaling, so insulin release is increased to maintain glucose homeostasis [1]. IR is associated with type 2 diabetes (T2D) or other metabolic risks such as metabolic syndrome, cardiovascular disease, and obesity [2, 3] and it was shown that inflammatory mechanisms and pro-inflammatory mediators have been largely involved in the pathogenesis of IR [4] and other metabolic disorders like T2D [5]. In this case, several pro-inflammatory cytokines such as tumor necrosis factor-alpha (TNF-α) [6, 7] and interleukin-6 (IL-6) [8] that develop inflammation through the reactive oxygen species (ROS) generation and oxidative stress pathways, decisively induce IR in peripheral tissues and adipocytes, particularly in a case of obesity [9]. Even, gestational weight gain was shown to associate with decreased pancreatic beta-cell function and impaired glucose-insulin metabolism in overweight/ obese pregnant women [10]. However, IR can also develop in individuals with normal body weight [11]. Furthermore, it is also linked with insulin action on protein and lipid metabolism as well as vascular endothelial function and gene expression [12–14].

Homeostasis model assessment for insulin resistance (HOMA-IR) was first introduced by Turner of the University of Oxford research group in 1985, which provides an estimate of IR based on fasting glucose and insulin levels. A higher score of HOMA-IR indicates more severe IR. Compared to hyperinsulinemic-euglycemic glucose clamp as the gold standard, HOMA-IR has been validated as a surrogate marker of IR for clinical and large-scale epidemiological research [15]. Even though it still has its limitations in the early diagnosis of IR [16].

The recent advent of techniques for comprehensive metabolic analysis, often termed “metabolomics”, has provided fresh insights into metabolic disorders [17]. Metabolomics is an analytical approach for identifying and quantifying endogenous small-molecule metabolites (< 1,500 Da) [18]. Alteration in metabolomics profiles may facilitate the forecast of specific metabolic diseases with high accuracy and help understand related fundamental mechanisms as well as metabolic pathways [17, 19]. Moreover, it is beneficial to classify personalized “metabolic signatures” and make it possible to suggest tailored therapy [20]. Various metabolomics tools, such as nuclear magnetic resonance spectroscopy (NMR), ultra-performance liquid chromatography (UPLC), and tandem mass spectrometry (MS/MS), have been used to generate metabolic profiles from blood, urine, or tissues [16].

Prevailing theories for the pathogenesis of IR focus on the dysregulation of carbohydrate metabolism, fat metabolism, and protein metabolism [1]. Among these, branched-chain amino acids (BCAAs), aromatic amino acids (AAAs), and acylcarnitines were reported to be associated with IR as biomarkers most commonly [11, 16], particularly BCAAs (leucine, isoleucine, and valine) and AAAs (phenylalanine and tyrosine). Also, comprehensive lipid profiling demonstrated that fatty acids of shorter carbon chain length and lower double bond content were associated with IR [21, 22]. Furthermore, IR could be caused by metabolic perturbations of fatty acid oxidation (FAO) and mitochondrial dysfunction [23]. Since the intracellular accumulation of acyl-CoA derivatives has been implicated in the development of IR, several studies showed its treatment with carnitine supplementation. Carnitine is a critical transporter of long-chain fatty acids in mitochondria, and its deficiency will impair the use of fat as fuel [24, 25]. The strongest associations were found for medium-chain acylcarnitines and IR [26]. Thus, accurate biomarkers or parameters that indicate IR state and metabolic risks are essential to understanding the pathophysiology of IR.

However, the true impact of these metabolites on IR, as well as the etiology and source of metabolite alterations, are unknown and incompletely understood, especially in nondiabetic individuals [22, 27, 28]. Additionally, studies have shown that Asians acquire IR at low BMIs [11], and even those with metabolic syndrome who do not fit the criteria for central obesity can develop IR [3, 29].

Here, we have measured and evaluated a wide array of metabolites (30 acylcarnitines and 20 amino acids) with the MS/MS method in a population-based study of 403 non-diabetic Iranian individuals and its association with IR.

## Materials and methods

The study population (*n* = 403) was chosen from non-diabetic individuals from participants of the Surveillance of Risk Factors of NCDs in Iran Study (STEPS 2016) who had sufficient data on their metabolites profile. STEPS 2016 is a population-based cross-sectional study conducted periodically on adults aged 18–75 years in 30 provinces of Iran to investigate none communicable risk factors [30]. After checking medical history via questionnaires and laboratory results, all subjects with a history of diabetes mellitus, using anti-diabetic medications, potential undiagnosed diabetes (defined by HbA1c ≥ 6.5% or fasting glucose ≥ 100 mg/dL), and who had incomplete data about their metabolite concentration were excluded.

### Blood sampling and biochemical measurement

Fasting venous blood samples were drawn into EDTA-containing tubes as well as sodium fluoride tubes. After the separation of plasma, the samples were stored at the appropriate temperature until analysis. Biochemical analytes including fasting plasma glucose (FPG), total cholesterol (TC), high-density lipoprotein cholesterol (HDL-C), triglycerides (TG), liver enzymes, uric acid, fructosamine, and HbA1c were measured by Cobas C311 auto analyzer using commercial kits from Roche Company (Roche Diagnostics, Mannheim, Germany). Insulin concentration was determined by the Electrochemiluminescence immunoassay method using Cobas e411 immunoanalyser from Roche Company.

Homeostatic Model Assessment for IR (HOMA-IR) values was calculated using the following formula: fasting insulin (µU/L) × fasting glucose (mg/dL)/405.

### Metabolomics analysis

Plasma concentration of amino acids and acylcarnitines were quantified by a targeted metabolomics analysis using flow‐injection tandem mass spectrometry (triple quadrupole API 3200 SCIEX with electrospray ionization) accompanied by Thermo Scientific Dionex UltiMate 3000 standard HPLC system. The analysis method, including preparation, derivatization, and validation, is published elsewhere [31, 32]. Briefly, 10 µL of samples/ calibrators/ quality control material were mixed with internal standard, and after centrifuging at 4˚C, supernatant fluids were separated and dried by a flow of nitrogen (99.9%) at 45˚C. In the next step, the samples were derivatized by acetyl chloride and 1-Butanol followed by incubation at 65˚C for 15 min. Finally, the samples were dried again by a flow of nitrogen (99.9%) and dissolved by 100 µL of acetonitrile and water.

### Statistical analysis

The metabolic syndrome was diagnosed according to the NCEP ATP III definition [33]. Based on this definition, the study participants were identified as having metabolic syndrome if they had three or more of the following features: 1) waist circumference ≥ 102 cm in men and ≥ 88 cm in women; 2) TGs ≥ 150 mg/dl; 3) FBS ≥ 100 mg/dl (or diagnosed diabetes); 4) HDL-C < 40 mg/dl in men and < 50 mg/dl in women; and 5) systolic blood pressure ≥ 130 mmHg or diastolic blood pressure ≥ 85 mmHg. The HOMA-IR cut-off point in the detection of metabolic syndrome was determined using the Yuden index, and the participants were classified into two groups: HOMA-IR < cut-off and HOMA-IR ≥ cut-off.

The data were checked for normality using descriptive analysis and Kolmogorov–Smirnov test. In each group, continuous variables were presented as mean ± SD and median (IQR) depending on the variables' normality. Categorical variables were expressed as numbers (%). Chi-Square and t-test were used to compare the results.

Univariate logistic regression was used to evaluate the correlation between metabolomics and HOMA-IR after the variables were standardized by Z value estimation. For multiple comparisons, obtained P-values were adjusted with the Benjamini–Hochberg method. Correlation between metabolomics variables was assessed by Pearson correlation, and a correlation matrix was provided. Considering the high correlation between metabolites, principal components analysis (PCA) was employed to make independent factors, in which the appropriateness was checked by Kaiser–Meyer–Olkin (KMO) and Bartlett sphericity tests. Factors were made based on eigenvalue more than 1 using varimax rotation with maximum likelihood [34]. A factor score was calculated for each individual based on the linear combination of amino acids and acylcarnitine and the loading factor for each component. The relationship between each component and the level of HOMA-IR was evaluated by logistic regressions separately, which were adjusted for age, sex, and BMI. Finally, the relationship between significant components in separate analyses was evaluated and the HOMA-IR level was investigated by a multiple regression model. Standardization was applied for the score of the components in all the logistic regression models. The significance level for statistical tests was 0.05, and R software version 3.6.1 was used for data analysis.

## Results

The study comprised 403 people (mean age 54.63 ± 12.13 years). Of them, 197 (48.9%) were male, and 206 (51.1%) were female. The mean HOMA-IR among the participants was 2.09 ± 1.58, and 118 (29.3%) of the subjects had metabolic syndrome. The HOMA-IR optimal cut-off was 1.95 based on the Yuden index to detect people with metabolic syndrome. Thus, 180 patients (44.7%) had a HOMA-IR equal to or more than 1.95, and 223 patients (55.3%) had a HOMA-IR less than 1.95. Sensitivity, specificity, PPV and NPV of this cut off point in IR detection were 68.4% (95% CI: 59.46% -76.87%), 65.26% (95% CI: 59.42% -70.78%), 45% (95% CI: 37.59% -52.58%) and 83.41% (95% CI: 77.86%—88.04%), respectively. The basic characteristics of the subjects in terms of HOMA-IR are presented in Table 1. A higher proportion of those with low IR were men (57.8% vs. 42.2%). People with high IR were significantly younger and had higher education level, BMI, waist circumference, FPG, HbA1c, ALT, TG, cholesterol, non-HDL cholesterol, uric acid, and a lower mean of HDL-C.

**Table 1:** Baseline characteristics of study population according to HOMA-IR level

Variable		HOMA-IR level		*P*-value
		< 1.95 (*n* = 223)	≥ 1.95 (*n* = 180)	
Gender, N (%)	Female	93 (41.7)	104 (57.8)	0.001
	Male	130 (58.3)	76 (42.2)	
Age (year)		56.80 ± 13.28	51.94 ± 9.94	< 0.001
BMI (kg/m^2^)		25.05 ± 4.56	26.17 ± 4.43	< 0.001
WC (cm)		88.57 ± 11.84	98.60 ± 10.89	< 0.001
HC (cm)		97.6 ± 10.4	105.8 ± 8.9	< 0.001
SBP (mmHg)		129.3 ± 21.19	128.8 ± 18.56	0.81
DBP (mmHg)		79.26 ± 11.82	80.77 ± 11.14	0.195
Education years, N (%)				0.015
< 1 year		47 (33.2)	38 (21.1)	
1–7 years		76 (34.1)	58 (32.2)	
8–12 years		48 (21.5)	57 (31.7)	
> 12 years		25 (11.2)	27 (15.0)	
FPG (mg/dL)		85.5 ± 9.3	90.2 ± 6.9	< 0.001
HbA1c (%)		5.41 ± 0.387	5.57 ± 0.337	< 0.001
Fructosamine (µmol/L)		233 ± 19	234 ± 25	0.81
TG (mg/dL)		104.1 ± 52.42	157.3 ± 88.86	< 0.001
Chol (mg/dL)		162.2 ± 33.54	174.1 ± 34.60	0.001
HDL-C (mg/dL)		44.56 ± 11.36	39.14 ± 11.13	< 0.001
Non-HDL Chol (mg/dL)		117.7 ± 33.77	134.9 ± 34.25	< 0.001
ALT (IU/L)		17.79 ± 9.32	23.46 ± 13.02	< 0.001
AST (IU/L)		22.74 ± 9.12	23.41 ± 7.84	0.43
Uric acid (mg/dL)		4.82 ± 1.20	5.42 ± 1.40	< 0.001

Continuous variables are presented as mean ± SD and categorical variables as Number (%)

*BMI* Body mass index, *WC* Waist circumference, *HC* Hip circumference, *SBP* Systolic blood pressure, *DBP* Diastolic blood pressure, *FPG* Fasting plasma glucose, *TG* Triglycerides, *Chol* Cholesterol, *HDL-C* High-density cholesterol, *ALT* Alanine aminotransferase

Table 2 shows the characteristics of individuals' metabolites by the level of IR. Table 3 shows the association between metabolites and IR for each metabolite, with or without adjusting the effect of age, sex, and BMI. After adjusting for age, sex, and BMI, among acylcarnitines, a higher C0 and C18: 1 respectively increased and decreased the chance of IR. Also, the higher levels of amino acids, including alanine, leucine, phenylalanine, valine, and tryptophan were associated with a greater chance of IR.

**Table 2:** Metabolites concentration according to HOMA-IR level

Acylcarnitines (µmol/L)	HOMA-IR level		Amino acids (µmol/L)	HOMA-IR level	
	< 1.95	≥ 1.95		< 1.95	≥ 1.95
C0	54.30 ± 13.47	57.61 ± 13.02	Alanine	364.7 ± 94.59	432.6 ± 95.16
C2	13.62(11.18—16.84)	13.84(11.61—16.17)	Aspartic Acid	11.8(9.800—13.85)	12(9.600—14.00)
C3	0.735(0.551—0.963)	0.831(0.638 -1.06)	Glutamic Acid	64.65(57.97—71.57)	67.9(60.20 -75.20)
C3DC	0.074(0.053—0.102)	0.0665(0.054 -0.091)	Leucine	114.9 ± 24.02	127.8 ± 23.66
C4	0.382(0.286—0.500)	0.404(0.297—0.551)	Methionine	26.9(23.85—30.40)	27.7(24.90—32.70)
C4OH	0.049(0.036—0.066)	0.0465(0.037—0.064)	Phenylalanine	59.49 ± 12.56	63.76 ± 10.53
C4DC	0.063(0.051—0.079)	0.065(0.055—0.080)	Tyrosine	64.38 ± 12.76	74.18 ± 13.78
C5	0.199(0.152—0.259)	0.2175(0.169—0.268)	Valine	238.0 ± 46.06	266.5 ± 41.97
C5:1	0.03(0.025—0.044)	0.032(0.026 -0.041)	Arginine	67.56 ± 20.88	69.52 ± 18.74
C5OH	0.06(0.051—0.074)	0.06(0.051—0.070)	Citrulline	40.03 ± 10.91	36.78 ± 9.43
C5DC	0.306(0.235—0.429)	0.285(0.236—0.364)	Glycine	256.1(221.6—323.7)	239.7(204.6—313.0)
C6	0.152(0.112—0.227)	0.1545(0.109—0.218)	Ornithine	91.2(77.20—110.7)	89(75.40—105.5)
C8	0.25(0.165—0.377)	0.237(0.171—0.352)	Proline	241.3 ± 91.09	259.6 ± 77.52
C8:1	0.289(0.202—0.437)	0.3185(0.213—0.439)	Threonine	139.09 ± 38.38	141.33 ± 32.34
C10	0.337(0.229—0.550)	0.311(0.219 -0.491)	Serine	107.7 ± 31.73	99.59 ± 24.88
C10:1	0.333(0.235—0.488)	0.316(0.234—0.471)	Histidine	80.87 ± 13.69	83.29 ± 15.89
C12	0.141(0.098—0.196)	0.1225(0.092—0.179)	Lysine	170.7 ± 40.54	170.08 ± 40.09
C14	0.058(0.044—0.077)	0.051(0.041—0.068)	Tryptophan	66.24 ± 14.83	72.82 ± 16.16
C14:1	0.123(0.088—0.180)	0.107(0.078—0.153)	Asparagine	43.6(33.47—54.90)	38.7(30.30—49.90)
C14:2	0.097(0.069—0.135)	0.086(0.065—0.117)	Glutamine	489.9 ± 111.2	478.0 ± 110.0
C14OH	0.012(0.009—0.017)	0.012(0.009—0.015)			
C16	0.173(0.143—0.216)	0.1705(0.146—0.204)			
C16OH	0.011(0.008—0.014)	0.01(0.008—0.013)			
C16:1OH	0.016(0.013—0.023)	0.015(0.012—0.019)			
C16:1	0.045(0.034—0.061)	0.043(0.032—0.058)			
C18	0.066(0.055—0.088)	0.062(0.051—0.075)			
C18:1	0.184(0.144—0.232)	0.1685(0.140—0.212)			
C18OH	0.008(0.006—0.010)	0.008(0.006—0.010)			
C18:1OH	0.012(0.009—0.016)	0.012(0.009—0.014)			
C18:2OH	0.027(0.021—0.034)	0.026(0.021—0.033)

**Table 3:** Associations between metabolites and HOMA-IR level with and without adjustment for age, sex, and BMI

**Acylcarnitines**	**OR**^a^ **(95% CI)**	***P* value**^b^	***P* value**^c^	**Amino acids**	**OR**^a^** (95% CI)**	***P* value**^b^	***P* value**^c^
C0	**1.287 (1.051—1.576)**	**0.044**	**0.013**	**Alanine**	**2.149 (1.70—2.73)**	** < 0.001**	** < 0.001**
C2	0.924 (0.754—1.13)	0.569	0.774	**Aspartic Acid**	1.035 (0.85—1.26)	0.831	0.7662
C3	1.18 (0.966—1.45)	0.226	0.142	**Glutamic Acid**	1.163 (0.933—1.50)	0.320	0.3545
C3DC	0.849 (0.691—1.04)	0.236	0.838	**Leucine**	**1.75 (1.41—2.17)**	** < 0.000**	** < 0.000**
C4	1.14 (0.934—1.40)	0.333	0.380	**Methionine**	1.312 (1.07—1.61)	0.030	0.089
C4OH	0.892 (0.729—1.09)	0.398	0.431	**Phenylalanine**	**1.452 (1.18—1.79)**	** < 0.000**	** < 0.000**
C4DC	1.03 (0.843—1.25)	0.891	0.963	**Tyrosine**	**2.21 (1.74—2.81)**	** < 0.000**	** < 0.000**
C5	1.18 (0.963—1.43)	0.235	0.153	**Valine**	**1.96 (1.56—2.45)**	** < 0.000**	** < 0.000**
C5_1	1.07 (0.879—1.30)	0.629	0.537	**Arginine**	1.103 (0.905—1.35)	0.463	0.536
C5OH	0.999 (0.82—1.22)	0.989	0.774	**Citrulline**	0.721 (0.585—0.888)	0.013	0.766
C5DC	0.752 (0.61—0.928)	0.029	0.413	**Glycine**	0.844 (0.689—1.03)	0.226	0.146
C6	1.01 (0.831—1.23)	0.933	0.983	**Ornithine**	0.916 (0.751—1.12)	0.511	0.983
C8	0.983 (0.806—1.20)	0.908	0.983	**Proline**	1.243 (1.01—1.52)	0.090	0.153
C8:1	1.09 (0.899—1.33)	0.499	0.983	**Threonine**	1.064 (0.874—1.30)	0.652	0.983
C10	0.957 (0.783—1.17)	0.780	0.988	**Serine**	0.747 (0.605—0.922)	0.029	0.1400
C10:1	0.956 (0.779—1.18)	0.780	0.988	**Histidine**	1.179 (0.966—1.44)	0.226	0.192
C12	0.887 (0.725—1.09)	0.394	0.811	**Lysine**	0.984 (0.808—1.20)	0.908	0.766
C14	0.744 (0.599—0.923)	0.029	0.267	**Tryptophan**	**1.555 (1.26—1.93)**	** < 0.000**	** < 0.000**
C14:1	0.782 (0.635—0.965)	0.061	0.142	**Asparagine**	0.777 (0.63 – 1.00)	0.056	0.267
C14:2	0.854 (0.694—1.05)	0.252	0.413	**Glutamine**	0.897 (0.735—1.10)	0.416	0.983
C14OH	0.795 (0.647—0.979)	0.079	0.463				
C16	0.906 (0.742—1.11)	0.463	0.7662				
C16OH	0.858 (0.701—1.05)	0.252	0.983				
C16:1OH	0.727 (0.586—0.902)	0.022	0.267				
C16:1	0.871 (0.699—1.09)	0.362	0.413				
C18	0.717 (0.578—0.889)	0.013	0.146				
C18:1	**0.743 (0.596—0.926)**	**0.029**	**0.013**				
C18OH	0.89 (0.729—1.09)	0.398	0.988				
C18:1OH	0.753 (0.611—0.928)	0.029	0.153				
C18:2OH	0.98 (0.803—1.20)	0.908	0.983				

All *p* values are adjusted for multiple testing using the Benjamini–Hochberg false discovery rate

^a^OR is measured per SD as data were transformed into Z-scores after adjustment for age, sex, and BMI

^b^Un-adjusted *p*-value

^c^*P* value is adjusted for age, sex, and BMI

**Fig. 1:**
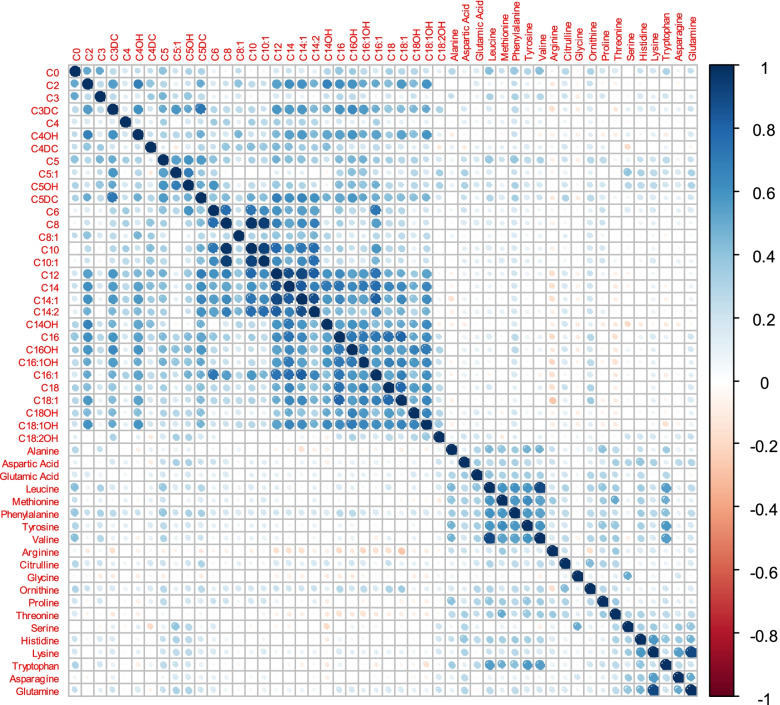
Collorogram of metabolites concentration correlation

Figure 1 shows the metabolite correlation matrix. The strongest correlations were found between leucine and valine (*r* = 0.907), C8 and C10 (*r* = 0.970), and C8 and C10: 1 (*r* = 0.943, Additional file 1).

According to PCA results, the sampling adequacy value with the KMO test was 0.867, and according to the Bartlett test, the correlation between metabolites was significant enough to perform PCA effectively (*p* < 0.001). Nine components were identified based on the Scree plot diagram (Additional file 2), accounting for 69.19% of the total variance. The findings for each component and its members are based on the maximal factor loading (Additional file 3). The first component includes C0, C4OH, C2, C5DC, C14, C14OH, C16, C16: 1, C16OH, C16: 1OH, C18OH, C18, C18: 1OH, and C18: 1, the second component includes C4, C4DC, C6, C8, C8: 1, C10, C10: 1, C12, C14: 1 and C14: 2, the third component includes leucine, tyrosine, valine, methionine, phenylalanine, alanine, glutamic acid, and threonine, the fourth component includes C3, C3DC, C5, C5OH, and C5: 1, the fifth component includes lysine, glutamine, asparagine, histidine, tryptophan, phenylalanine, alanine, glutamic acid, and threonine, the sixth component includes citrulline, ornithine, and proline, the seventh component contains arginine and aspartic acid, the eighth component includes glycine and serine, and the ninth component was C18: 2OH.

**Fig. 2:**
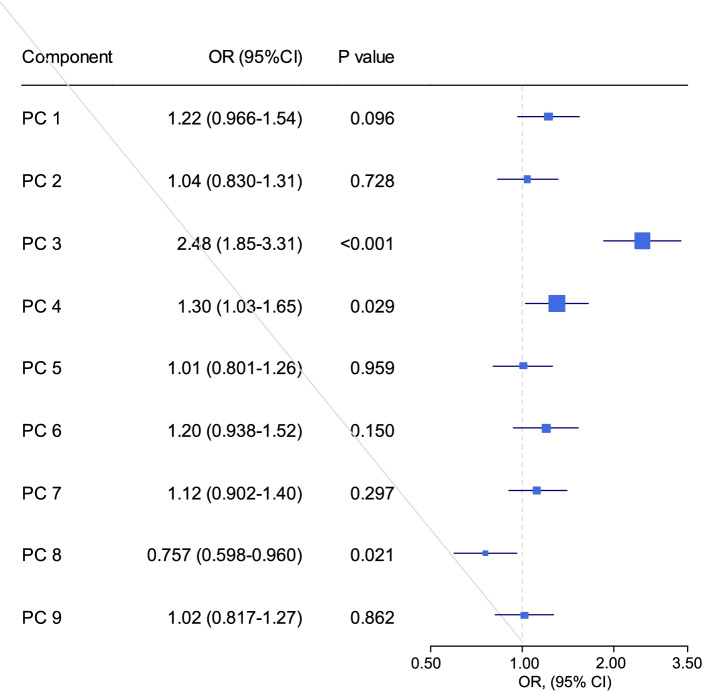
Association of components and HOMA-IR adjusted for age, sex, and BMI

Figure 2 depicts the association between each component and IR after adjusting for the effects of age, sex, and BMI. Components 3, 4, and 8 were significantly associated with IR, and since the loading of all amino acids in this component is positive, a higher amount of this component is associated with an increased chance of developing IR (OR = 2.48; *p* < 0.001). Loading of all metabolites in the fourth component was positive, so higher levels of this component were associated with an increased risk of developing IR (OR = 1.30; *p* = 0.029). The eighth component, including glycine and serine, had positive loading, and with increasing the amount of this component, the chances of IR decreased (OR = 0.757, *p* = 0.021). Multiple logistic regression findings revealed that when the effects of age, sex, BMI, and components were adjusted, the eighth and third components were substantial predictors of IR, but the fourth component lost its impact (Table 4).

**Table 4:** Multiple Logistic regression on the association of metabolomics factors with HOMA-IR level. *P* value was adjusted for age, sex, BMI and components

Components	Coefficient	SE	OR	95% CI	*P* value
PC 3	-0.408	0.132	2.74	2.00 – 3.75	< 0.001
PC 4	0.053	0.138	1.05	0.804 – 1.38	0.702
PC 8	1.01	0.161	0.665	0.513 -0.862	0.002

## Discussion

**Fig. 3:**
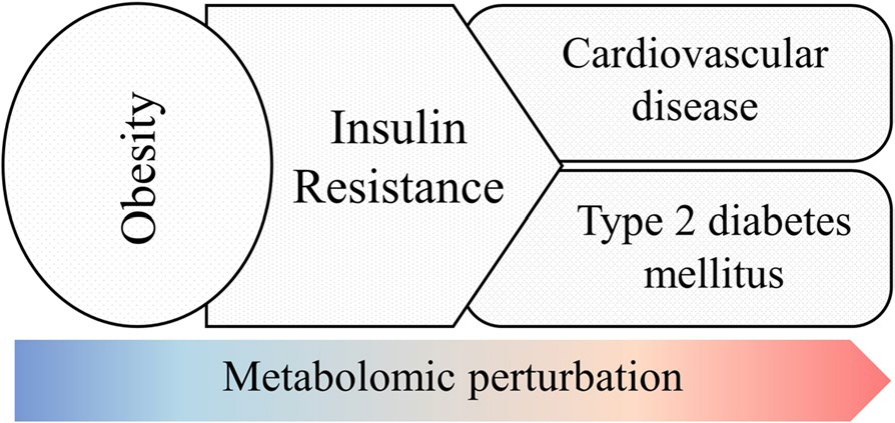
Association of insulin resistance with cardiovascular disease, type 2 diabetes mellitus, obesity and metabolomic perturbation

Excessive energy intake, especially from simple carbohydrate and total carbohydrate, and low energy expenditure caused the development of IR [35]. IR is a condition in which a particular concentration of insulin produces a less-than-expected biological effect; that makes up a broad clinical spectrum including diabetes, obesity, and metabolic syndrome, which will affect the patient's quality of life (Fig. 3) [25].

In this study, we included 403 non-diabetic patients from STEP2016. All metabolite measurements were quantified with the MS/MS-based metabolomics platform, which allowed for a comparison of the absolute concentrations across all individuals. The optimal cut-off point of HOMA-IR for the detection of IR in our study population was calculated to be 1.95.

The most noticeable finding is a strong positive association of serum BCAAs (valine and leucine), AAAs (tyrosine, tryptophan, and phenylalanine), alanine, and C0 (free carnitine) with IR (HOMA-IR); while C18:1 (oleoyl L-carnitine) was inversely correlated with IR. Elevation in the BCAAs and AAAs has been previously found in IR state and was strongly associated with early IR and T2D prediction regardless of BMI [19, 36, 37]. So, they are detectable about one decade before the onset of T2D, suggesting these metabolites might serve as potential biomarkers to predict future T2D [38–40].

The mechanism between BCAAs and IR is not completely understood [37, 41]. However, there is some evidence supporting this correlation. Insulin is a regulator of branched-chain α-ketoacid dehydrogenase (BCKD) complex, a rate-limiting enzyme of BCAA catabolism. Reduced enzyme activity and perturbation of the BCKD complex in hyperinsulinemia and diabetes have been observed [36, 42–44]. Also, findings suggest that the effects of leucine, but not other BCAAs, inhibit insulin-sensitive glucose uptake via the mTOR signaling pathway [45, 46]. Moreover, in BCAA dysmetabolism, some potentially toxic intermediates might be accumulated, resulting in impaired cellular or organ function [47]. Olson et al. found that toxic BCAA metabolites, rather than BCAAs themselves, can cause mitochondrial dysfunction [48].

Alanine also showed a positive association with HOMA-IR. Glutamate is produced in the first step of BCAA catabolism. Glutamate accumulation may increase the transamination of pyruvate to alanine, which may promote gluconeogenesis and therefore contribute to the development of glucose intolerance [22, 49]. Interestingly, because the alanine increase is dependent on BCAAs, its alteration is less significant than BCAAs and AAAs in our results.

Tryptophan levels tended to show a substantial association with IR. These results verified previous findings that tryptophan and its downstream metabolite serotonin (5-hydroxytryptamine), kynurenine, and xanthurenic acid play important roles in the regulation of IR, pancreatic beta-cell function, and glucose homeostasis [50]. Furthermore, tyrosine was discovered to be substantially linked to IR as well as obesity in children [51]. Tyrosine was also discovered to be a significant predictor of diabetes in males from South Asia [52]. Also, findings showed that AAAs are higher in obese compared to lean subjects. This is probably because of the “large neutral amino acids” (tryptophan, phenylalanine, tyrosine, leucine, isoleucine, valine), which include both BCAAs and AAAs, compete for transport into mammalian cells via the large neutral amino acid transporter (LAT1). Assuming that persistent BCAA increases limit AAA transfer into cells and tissues [22, 53].

Acetylcarnitine levels rise during acetyl-CoA overload which occurs when glycolysis or β-oxidation surpasses the tricarboxylic acid (TCA) cycle activity and has contributed to substrate switching and glucose homeostasis [54, 55]. When IR is present, free fatty acids overload β-oxidation on skeletal muscle and liver in an attempt to maintain energy substrate [19].

Long-chain acyl-CoA and other fatty acid metabolites accumulate in skeletal muscle and heart, impair insulin signaling and contribute to the development of IR [56, 57]. Furthermore, carnitine could modulate the intramitochondrial acetyl-CoA/CoA ratio and the activity of the pyruvate dehydrogenase complex (PDHC), alter the expression of glycolytic and gluconeogenic enzymes, stimulate the IGF-1 axis and IGF-1 signaling pathway, and alter the expression of insulin signaling pathway genes [24].

In the area of IR, the notion of lipotoxicity associated with dysfunctional β-oxidation is well acknowledged, and increasing emphasis has been paid to intramitochondrial changes and impairments in mitochondrial FAO, particularly on acylcarnitines [58, 59]. Furthermore, acylcarnitines may induce NF-κB signaling and cytokine production in macrophages and epithelial cells as well as alter insulin sensitivity through a proinflammatory response [4, 60] and in this case, anti-inflammatory therapy such as an interleukin-1 receptor antagonist showed improvement in insulin sensitivity [61]. Acylcarnitine rise could be a secondary manifestation of IR because of perturbation in mitochondrial function. However, acylcarnitine by itself could accentuate IR intensity with NF-κB signaling which could be a vicious circle. According to our results, the association of the BCAA and AAA cluster with IR was stronger than that observed from acylcarnitine clusters, which is supported by the Newgard et al. study as well [22]. This strengthens the secondary manifestation role of acylcarnitine in IR.

Dysregulation of the outer mitochondrial membrane enzyme carnitine palmitoyltransferase (CPT1) and mitochondrial matrix protein CPT2 with subsequent effects on energy production from FAO and impaired feedback regulation of glucose metabolism could underlie the alteration in acylcarnitines [62, 63]. In our study, IR was associated with an increase in C0 and a reduction in C18 which is comparable to clinical presentation of CPT1 deficiency disease [64].

The concept of increased, though incomplete, FAO by disproportional regulation of FAO, TCA cycle, and respiratory chain is attractive to explain IR. However, there remains doubt about this mechanism, and there is no proof that acylcarnitines play a role in the induction of IR itself. Acylcarnitines are present under physiological conditions, and their levels vary according to dietary circumstances [65]. Carnitine supplementation demonstrates an improvement in IR states. However, little heterogeneity existed in the studies [25]. Our factor analysis demonstrates that the eighth component consisted of glycine and serine inversely associated with IR. In line with our finding, Sekhar RV showed that glycine and N-acetylcysteine supplementation improves the impaired mitochondrial FAO and decreases IR [66].

Limitations of our study include unknown generalizability to ethnic groups causes IR-related biomarkers particularly, BCAAs seem to be race dependent due to the different genetic backgrounds [41]. Therefore, the valuable result of this study must be compared with other ethnic groups. Also, HOMA-IR might be considered an “imprecise” method for assessing IR. However, individuals from opposing HOMA-IR index categories had clear differences in the concentrations of TG and HDL-C, supporting the idea that these individuals had true differences in insulin sensitivity.

Novel markers may aid to elucidate aspects of metabolic dysfunction that contribute to diabetes risk and improve the early detection of this condition [41]. Because T2D has a progressive onset, metabolite profiles at multiple time points before T2D diagnosis would be useful to identify new, early diagnostic biomarkers of T2D [67]. In the present study, we identified specific metabolites linked to HOMA-IR that improved IR prediction. However, further studies are required to target the related metabolites with therapeutic strategies and assess their power in predictive risk scores. To associate metabolites in a predictive matter, observations must be performed prior to the development of disease to determine the risk of disease and comorbidities. In conclusion, our study adds more evidence that a specific metabolomic profile is associated with insulin resistance and reemphasizes the significance of understanding the biochemistry and physiology which lead to these associations.

## Supplementary Information

**Additional file 1.** The correlation coefficient estimates between metabolites.

**Additional file 2.** Scree plot resulted from factor analysis.

**Additional file 3.** Factor loadings of PCA considering Varimax rotation.

## Data Availability

The dataset analyzed during the current study is not publicly available because we obtained our dataset from Iran STEPS Noncommunicable Disease Risk Factors Survey 2016 under the Material Transfer agreement; however, the corresponding author (Dr. Farideh Razi) will facilitate communication with the chief investigator of STEPs 2016 if private data is needed on reasonable request.
