# Supplementary material for: Metabolic signatures of insulin resistance in non-diabetic individuals
Source: BMC Endocr Disord. 2022 Aug 24;22:212. doi: 10.1186/s12902-022-01130-3 (PMC9404631; doi:10.1186/s12902-022-01130-3)
Supplement: Supplementary file 1 — Additional file 1. The correlation coefficient estimates between metabolites. [file 12902_2022_1130_MOESM1_ESM.docx]

Additional file 1. The correlation coefficient estimates between metabolites

|  | C0 | C2 | C3 | C3DC | C4 | C4OH | C4DC | C5 | C5:1 | C5OH |
| --- | --- | --- | --- | --- | --- | --- | --- | --- | --- | --- |
| C0 | 1.00 | 0.52 | 0.52 | 0.27 | 0.16 | 0.25 | 0.24 | 0.40 | 0.21 | 0.30 |
| C2 | 0.52 | 1.00 | 0.26 | 0.56 | 0.17 | 0.69 | 0.39 | 0.29 | 0.11 | 0.21 |
| C3 | 0.52 | 0.26 | 1.00 | 0.27 | 0.15 | 0.16 | 0.37 | 0.46 | 0.25 | 0.38 |
| C3DC | 0.27 | 0.56 | 0.27 | 1.00 | 0.15 | 0.61 | 0.20 | 0.51 | 0.59 | 0.52 |
| C4 | 0.16 | 0.17 | 0.15 | 0.15 | 1.00 | 0.21 | 0.13 | 0.34 | 0.03 | 0.17 |
| C4OH | 0.25 | 0.69 | 0.16 | 0.61 | 0.21 | 1.00 | 0.22 | 0.31 | 0.27 | 0.30 |
| C4DC | 0.24 | 0.39 | 0.37 | 0.20 | 0.13 | 0.22 | 1.00 | 0.15 | -0.13 | 0.16 |
| C5 | 0.40 | 0.29 | 0.46 | 0.51 | 0.34 | 0.31 | 0.15 | 1.00 | 0.55 | 0.59 |
| C5:1 | 0.21 | 0.11 | 0.25 | 0.59 | 0.03 | 0.27 | -0.13 | 0.55 | 1.00 | 0.69 |
| C5OH | 0.30 | 0.21 | 0.38 | 0.52 | 0.17 | 0.30 | 0.16 | 0.59 | 0.69 | 1.00 |
| C5DC | 0.31 | 0.51 | 0.25 | 0.73 | 0.24 | 0.50 | 0.30 | 0.56 | 0.37 | 0.51 |
| C6 | 0.08 | 0.16 | 0.09 | 0.30 | 0.36 | 0.22 | 0.22 | 0.41 | 0.28 | 0.60 |
| C8 | 0.06 | 0.21 | 0.09 | 0.25 | 0.33 | 0.18 | 0.40 | 0.33 | 0.05 | 0.29 |
| C8:1 | 0.25 | 0.41 | 0.19 | 0.26 | 0.14 | 0.47 | 0.35 | 0.18 | -0.01 | 0.11 |
| C10 | 0.05 | 0.26 | 0.06 | 0.29 | 0.34 | 0.22 | 0.43 | 0.33 | 0.00 | 0.25 |
| C10:1 | 0.05 | 0.22 | 0.05 | 0.23 | 0.27 | 0.21 | 0.38 | 0.26 | -0.02 | 0.17 |
| C12 | 0.19 | 0.55 | 0.18 | 0.58 | 0.35 | 0.46 | 0.45 | 0.48 | 0.14 | 0.38 |
| C14 | 0.23 | 0.62 | 0.19 | 0.56 | 0.27 | 0.55 | 0.44 | 0.43 | 0.14 | 0.37 |
| C14:1 | 0.15 | 0.61 | 0.09 | 0.59 | 0.32 | 0.57 | 0.35 | 0.41 | 0.12 | 0.31 |
| C14:2 | 0.13 | 0.48 | 0.06 | 0.45 | 0.28 | 0.45 | 0.35 | 0.32 | 0.01 | 0.22 |
| C14OH | 0.29 | 0.67 | 0.23 | 0.48 | 0.14 | 0.53 | 0.44 | 0.22 | -0.05 | 0.06 |
| C16 | 0.39 | 0.62 | 0.29 | 0.59 | 0.19 | 0.54 | 0.29 | 0.44 | 0.30 | 0.45 |
| C16OH | 0.36 | 0.59 | 0.25 | 0.64 | 0.15 | 0.59 | 0.19 | 0.46 | 0.43 | 0.45 |
| C16:1OH | 0.25 | 0.52 | 0.20 | 0.56 | 0.11 | 0.51 | 0.17 | 0.49 | 0.39 | 0.39 |
| C16:1 | 0.18 | 0.52 | 0.07 | 0.49 | 0.33 | 0.53 | 0.26 | 0.36 | 0.24 | 0.49 |
| C18 | 0.33 | 0.43 | 0.20 | 0.39 | 0.11 | 0.36 | 0.22 | 0.34 | 0.14 | 0.29 |
| C18:1 | 0.28 | 0.58 | 0.10 | 0.43 | 0.14 | 0.53 | 0.19 | 0.27 | 0.15 | 0.24 |
| C18OH | 0.29 | 0.45 | 0.23 | 0.45 | 0.07 | 0.43 | 0.15 | 0.30 | 0.29 | 0.27 |
| C18:1OH | 0.22 | 0.61 | 0.08 | 0.52 | 0.13 | 0.60 | 0.20 | 0.25 | 0.17 | 0.25 |
| C18:2OH | 0.13 | 0.03 | 0.04 | 0.32 | 0.00 | 0.13 | -0.14 | 0.17 | 0.34 | 0.31 |
| Alanine | 0.28 | -0.01 | 0.24 | -0.02 | -0.02 | -0.10 | 0.11 | 0.08 | 0.09 | 0.11 |
| Aspartic Acid | 0.11 | -0.01 | 0.05 | 0.18 | 0.13 | 0.05 | -0.12 | 0.24 | 0.33 | 0.32 |
| Glutamic Acid | 0.16 | 0.15 | 0.09 | 0.03 | 0.07 | -0.02 | 0.12 | 0.18 | 0.09 | 0.18 |
| Leucine | 0.42 | 0.09 | 0.32 | 0.17 | 0.13 | -0.03 | 0.11 | 0.38 | 0.15 | 0.24 |
| Methionine | 0.23 | 0.07 | 0.13 | 0.05 | 0.10 | -0.05 | 0.13 | 0.21 | -0.01 | 0.09 |
| Phenylalanine | 0.38 | 0.24 | 0.31 | 0.27 | 0.19 | 0.23 | 0.15 | 0.39 | 0.22 | 0.32 |
| Tyrosine | 0.30 | 0.11 | 0.20 | 0.08 | 0.15 | 0.01 | 0.16 | 0.26 | 0.12 | 0.20 |
| Valine | 0.42 | 0.07 | 0.28 | 0.11 | 0.12 | -0.08 | 0.11 | 0.31 | 0.10 | 0.18 |
| Arginine | 0.07 | -0.13 | 0.07 | -0.17 | 0.02 | -0.08 | 0.06 | -0.09 | -0.07 | -0.04 |
| Citrulline | 0.28 | 0.16 | 0.21 | 0.15 | 0.07 | 0.09 | 0.18 | 0.21 | 0.08 | 0.20 |
| Glycine | -0.04 | -0.05 | -0.12 | -0.13 | -0.01 | -0.09 | -0.05 | -0.11 | 0.01 | 0.05 |
| Ornithine | 0.31 | 0.22 | 0.15 | 0.16 | 0.08 | 0.14 | 0.17 | 0.27 | 0.13 | 0.22 |
| Proline | 0.20 | -0.06 | 0.19 | -0.02 | 0.05 | -0.10 | 0.12 | 0.25 | 0.02 | 0.19 |
| Threonine | 0.16 | -0.07 | 0.02 | -0.14 | 0.03 | -0.10 | -0.01 | 0.06 | -0.02 | 0.02 |
| Serine | 0.00 | -0.02 | -0.05 | 0.14 | -0.04 | 0.15 | -0.20 | 0.11 | 0.40 | 0.27 |
| Histidine | 0.21 | 0.05 | 0.17 | 0.15 | 0.02 | 0.06 | -0.01 | 0.24 | 0.31 | 0.26 |
| Lysine | 0.18 | 0.00 | 0.13 | 0.17 | 0.02 | 0.14 | 0.00 | 0.21 | 0.25 | 0.27 |
| Tryptophan | 0.30 | 0.02 | 0.21 | 0.11 | 0.02 | -0.10 | 0.09 | 0.26 | 0.23 | 0.20 |
| Asparagine | 0.07 | -0.05 | 0.01 | 0.10 | -0.02 | 0.02 | 0.02 | 0.07 | 0.17 | 0.16 |
| Glutamine | 0.25 | -0.02 | 0.15 | 0.22 | 0.02 | 0.16 | -0.06 | 0.23 | 0.31 | 0.31 |
|  | C10:1 | C12 | C14 | C14:1 | C14:2 | C14OH | C16 | C16OH | C16:1OH | C16:1 |
| C0 | 0.05 | 0.19 | 0.23 | 0.15 | 0.13 | 0.29 | 0.39 | 0.36 | 0.25 | 0.18 |
| C2 | 0.22 | 0.55 | 0.62 | 0.61 | 0.48 | 0.67 | 0.62 | 0.59 | 0.52 | 0.52 |
| C3 | 0.05 | 0.18 | 0.19 | 0.09 | 0.06 | 0.23 | 0.29 | 0.25 | 0.20 | 0.07 |
| C3DC | 0.23 | 0.58 | 0.56 | 0.59 | 0.45 | 0.48 | 0.59 | 0.64 | 0.56 | 0.49 |
| C4 | 0.27 | 0.35 | 0.27 | 0.32 | 0.28 | 0.14 | 0.19 | 0.15 | 0.11 | 0.33 |
| C4OH | 0.21 | 0.46 | 0.55 | 0.57 | 0.45 | 0.53 | 0.54 | 0.59 | 0.51 | 0.53 |
| C4DC | 0.38 | 0.45 | 0.44 | 0.35 | 0.35 | 0.44 | 0.29 | 0.19 | 0.17 | 0.26 |
| C5 | 0.26 | 0.48 | 0.43 | 0.41 | 0.32 | 0.22 | 0.44 | 0.46 | 0.49 | 0.36 |
| C5:1 | -0.02 | 0.14 | 0.14 | 0.12 | 0.01 | -0.05 | 0.30 | 0.43 | 0.39 | 0.24 |
| C5OH | 0.17 | 0.38 | 0.37 | 0.31 | 0.22 | 0.06 | 0.45 | 0.45 | 0.39 | 0.49 |
| C5DC | 0.43 | 0.69 | 0.61 | 0.66 | 0.60 | 0.49 | 0.53 | 0.58 | 0.52 | 0.50 |
| C6 | 0.62 | 0.59 | 0.50 | 0.56 | 0.53 | 0.02 | 0.39 | 0.26 | 0.25 | 0.73 |
| C8 | 0.94 | 0.68 | 0.52 | 0.64 | 0.69 | 0.17 | 0.33 | 0.22 | 0.23 | 0.55 |
| C8:1 | 0.35 | 0.39 | 0.37 | 0.39 | 0.46 | 0.29 | 0.19 | 0.16 | 0.14 | 0.29 |
| C10 | 0.92 | 0.78 | 0.60 | 0.72 | 0.77 | 0.27 | 0.36 | 0.25 | 0.27 | 0.58 |
| C10:1 | 1.00 | 0.63 | 0.47 | 0.63 | 0.76 | 0.20 | 0.26 | 0.18 | 0.16 | 0.47 |
| C12 | 0.63 | 1.00 | 0.89 | 0.90 | 0.83 | 0.60 | 0.63 | 0.54 | 0.56 | 0.73 |
| C14 | 0.47 | 0.89 | 1.00 | 0.83 | 0.68 | 0.72 | 0.75 | 0.64 | 0.72 | 0.77 |
| C14:1 | 0.63 | 0.90 | 0.83 | 1.00 | 0.89 | 0.59 | 0.65 | 0.56 | 0.55 | 0.82 |
| C14:2 | 0.76 | 0.83 | 0.68 | 0.89 | 1.00 | 0.46 | 0.45 | 0.38 | 0.34 | 0.65 |
| C14OH | 0.20 | 0.60 | 0.72 | 0.59 | 0.46 | 1.00 | 0.55 | 0.60 | 0.60 | 0.43 |
| C16 | 0.26 | 0.63 | 0.75 | 0.65 | 0.45 | 0.55 | 1.00 | 0.71 | 0.71 | 0.72 |
| C16OH | 0.18 | 0.54 | 0.64 | 0.56 | 0.38 | 0.60 | 0.71 | 1.00 | 0.71 | 0.58 |
| C16:1OH | 0.16 | 0.56 | 0.72 | 0.55 | 0.34 | 0.60 | 0.71 | 0.71 | 1.00 | 0.57 |
| C16:1 | 0.47 | 0.73 | 0.77 | 0.82 | 0.65 | 0.43 | 0.72 | 0.58 | 0.57 | 1.00 |
| C18 | 0.21 | 0.51 | 0.66 | 0.50 | 0.39 | 0.50 | 0.76 | 0.58 | 0.69 | 0.53 |
| C18:1 | 0.25 | 0.51 | 0.64 | 0.64 | 0.48 | 0.52 | 0.80 | 0.56 | 0.67 | 0.69 |
| C18OH | 0.09 | 0.36 | 0.50 | 0.40 | 0.28 | 0.52 | 0.52 | 0.68 | 0.58 | 0.41 |
| C18:1OH | 0.22 | 0.53 | 0.65 | 0.62 | 0.48 | 0.65 | 0.62 | 0.71 | 0.66 | 0.61 |
| C18:2OH | 0.01 | 0.04 | 0.04 | 0.08 | 0.06 | -0.08 | 0.25 | 0.31 | 0.22 | 0.17 |
| Alanine | 0.02 | -0.07 | -0.08 | -0.16 | -0.09 | -0.09 | 0.02 | 0.02 | -0.07 | -0.08 |
| Aspartic Acid | 0.15 | 0.08 | 0.02 | 0.08 | 0.11 | -0.10 | 0.08 | 0.10 | 0.08 | 0.10 |
| Glutamic Acid | 0.14 | 0.12 | 0.11 | 0.07 | 0.11 | -0.01 | 0.13 | 0.12 | 0.10 | 0.06 |
| Leucine | 0.14 | 0.15 | 0.08 | 0.04 | 0.12 | 0.04 | 0.15 | 0.13 | 0.05 | -0.05 |
| Methionine | 0.15 | 0.10 | 0.04 | 0.03 | 0.13 | 0.04 | 0.05 | -0.03 | -0.02 | -0.02 |
| Phenylalanine | 0.18 | 0.20 | 0.20 | 0.16 | 0.19 | 0.16 | 0.27 | 0.27 | 0.20 | 0.17 |
| Tyrosine | 0.14 | 0.09 | 0.03 | 0.04 | 0.09 | 0.00 | 0.10 | 0.07 | 0.01 | 0.04 |
| Valine | 0.11 | 0.10 | 0.04 | -0.01 | 0.07 | 0.02 | 0.12 | 0.10 | -0.02 | -0.08 |
| Arginine | -0.05 | -0.17 | -0.18 | -0.17 | -0.10 | -0.10 | -0.20 | -0.14 | -0.22 | -0.15 |
| Citrulline | 0.08 | 0.17 | 0.17 | 0.13 | 0.18 | 0.20 | 0.13 | 0.19 | 0.15 | 0.05 |
| Glycine | -0.02 | -0.08 | -0.09 | -0.08 | -0.03 | -0.14 | -0.12 | -0.14 | -0.06 | -0.03 |
| Ornithine | 0.18 | 0.23 | 0.21 | 0.21 | 0.23 | 0.13 | 0.29 | 0.20 | 0.26 | 0.16 |
| Proline | 0.21 | 0.08 | 0.02 | 0.05 | 0.13 | -0.11 | 0.00 | -0.03 | -0.07 | 0.02 |
| Threonine | 0.07 | -0.08 | -0.14 | -0.11 | 0.02 | -0.15 | -0.17 | -0.14 | -0.16 | -0.15 |
| Serine | 0.00 | -0.07 | -0.07 | 0.00 | 0.00 | -0.20 | 0.00 | 0.08 | 0.05 | 0.06 |
| Histidine | 0.08 | 0.02 | -0.01 | -0.01 | 0.03 | -0.12 | 0.09 | 0.05 | 0.06 | 0.01 |
| Lysine | 0.16 | 0.06 | 0.09 | 0.09 | 0.08 | -0.04 | 0.17 | 0.14 | 0.11 | 0.13 |
| Tryptophan | 0.02 | 0.01 | -0.06 | -0.08 | -0.04 | -0.14 | 0.10 | 0.01 | -0.02 | -0.12 |
| Asparagine | 0.10 | 0.01 | -0.01 | 0.01 | 0.04 | -0.11 | 0.03 | 0.01 | -0.02 | 0.03 |
| Glutamine | 0.10 | 0.06 | 0.09 | 0.09 | 0.08 | -0.03 | 0.20 | 0.19 | 0.15 | 0.14 |
|  | C18 | C18:1 | C18OH | C18:1OH | C18:2OH | Alanine | Aspartic Acid | Glutamic Acid | Leucine | Methionine |
| C0 | 0.33 | 0.28 | 0.29 | 0.22 | 0.13 | 0.28 | 0.11 | 0.16 | 0.42 | 0.23 |
| C2 | 0.43 | 0.58 | 0.45 | 0.61 | 0.03 | -0.01 | -0.01 | 0.15 | 0.09 | 0.07 |
| C3 | 0.20 | 0.10 | 0.23 | 0.08 | 0.04 | 0.24 | 0.05 | 0.09 | 0.32 | 0.13 |
| C3DC | 0.39 | 0.43 | 0.45 | 0.52 | 0.32 | -0.02 | 0.18 | 0.03 | 0.17 | 0.05 |
| C4 | 0.11 | 0.14 | 0.07 | 0.13 | 0.00 | -0.02 | 0.13 | 0.07 | 0.13 | 0.10 |
| C4OH | 0.36 | 0.53 | 0.43 | 0.60 | 0.13 | -0.10 | 0.05 | -0.02 | -0.03 | -0.05 |
| C4DC | 0.22 | 0.19 | 0.15 | 0.20 | -0.14 | 0.11 | -0.12 | 0.12 | 0.11 | 0.13 |
| C5 | 0.34 | 0.27 | 0.30 | 0.25 | 0.17 | 0.08 | 0.24 | 0.18 | 0.38 | 0.21 |
| C5:1 | 0.14 | 0.15 | 0.29 | 0.17 | 0.34 | 0.09 | 0.33 | 0.09 | 0.15 | -0.01 |
| C5OH | 0.29 | 0.24 | 0.27 | 0.25 | 0.31 | 0.11 | 0.32 | 0.18 | 0.24 | 0.09 |
| C5DC | 0.41 | 0.43 | 0.41 | 0.47 | 0.17 | 0.00 | 0.23 | 0.12 | 0.30 | 0.15 |
| C6 | 0.29 | 0.28 | 0.10 | 0.21 | 0.11 | 0.06 | 0.22 | 0.11 | 0.10 | 0.10 |
| C8 | 0.25 | 0.27 | 0.09 | 0.22 | 0.03 | 0.03 | 0.16 | 0.16 | 0.15 | 0.15 |
| C8:1 | 0.20 | 0.25 | 0.17 | 0.27 | -0.03 | 0.08 | -0.05 | 0.02 | 0.08 | 0.05 |
| C10 | 0.29 | 0.30 | 0.12 | 0.27 | 0.00 | -0.01 | 0.13 | 0.14 | 0.15 | 0.13 |
| C10:1 | 0.21 | 0.25 | 0.09 | 0.22 | 0.01 | 0.02 | 0.15 | 0.14 | 0.14 | 0.15 |
| C12 | 0.51 | 0.51 | 0.36 | 0.53 | 0.04 | -0.07 | 0.08 | 0.12 | 0.15 | 0.10 |
| C14 | 0.66 | 0.64 | 0.50 | 0.65 | 0.04 | -0.08 | 0.02 | 0.11 | 0.08 | 0.04 |
| C14:1 | 0.50 | 0.64 | 0.40 | 0.62 | 0.08 | -0.16 | 0.08 | 0.07 | 0.04 | 0.03 |
| C14:2 | 0.39 | 0.48 | 0.28 | 0.48 | 0.06 | -0.09 | 0.11 | 0.11 | 0.12 | 0.13 |
| C14OH | 0.50 | 0.52 | 0.52 | 0.65 | -0.08 | -0.09 | -0.10 | -0.01 | 0.04 | 0.04 |
| C16 | 0.76 | 0.80 | 0.52 | 0.62 | 0.25 | 0.02 | 0.08 | 0.13 | 0.15 | 0.05 |
| C16OH | 0.58 | 0.56 | 0.68 | 0.71 | 0.31 | 0.02 | 0.10 | 0.12 | 0.13 | -0.03 |
| C16:1OH | 0.69 | 0.67 | 0.58 | 0.66 | 0.22 | -0.07 | 0.08 | 0.10 | 0.05 | -0.02 |
| C16:1 | 0.53 | 0.69 | 0.41 | 0.61 | 0.17 | -0.08 | 0.10 | 0.06 | -0.05 | -0.02 |
| C18 | 1.00 | 0.79 | 0.56 | 0.61 | 0.26 | 0.00 | 0.07 | 0.12 | 0.18 | -0.01 |
| C18:1 | 0.79 | 1.00 | 0.49 | 0.66 | 0.20 | -0.11 | 0.05 | 0.04 | 0.01 | -0.05 |
| C18OH | 0.56 | 0.49 | 1.00 | 0.66 | 0.28 | 0.08 | 0.08 | 0.16 | 0.14 | -0.06 |
| C18:1OH | 0.61 | 0.66 | 0.66 | 1.00 | 0.38 | -0.09 | 0.06 | 0.12 | 0.03 | -0.05 |
| C18:2OH | 0.26 | 0.20 | 0.28 | 0.38 | 1.00 | 0.08 | 0.20 | 0.19 | 0.16 | -0.12 |
| Alanine | 0.00 | -0.11 | 0.08 | -0.09 | 0.08 | 1.00 | 0.15 | 0.30 | 0.46 | 0.40 |
| Aspartic Acid | 0.07 | 0.05 | 0.08 | 0.06 | 0.20 | 0.15 | 1.00 | 0.37 | 0.26 | 0.32 |
| Glutamic Acid | 0.12 | 0.04 | 0.16 | 0.12 | 0.19 | 0.30 | 0.37 | 1.00 | 0.40 | 0.31 |
| Leucine | 0.18 | 0.01 | 0.14 | 0.03 | 0.16 | 0.46 | 0.26 | 0.40 | 1.00 | 0.59 |
| Methionine | -0.01 | -0.05 | -0.06 | -0.05 | -0.12 | 0.40 | 0.32 | 0.31 | 0.59 | 1.00 |
| Phenylalanine | 0.14 | 0.12 | 0.20 | 0.18 | 0.15 | 0.35 | 0.33 | 0.33 | 0.61 | 0.59 |
| Tyrosine | 0.01 | -0.02 | 0.06 | 0.01 | 0.02 | 0.47 | 0.25 | 0.35 | 0.58 | 0.63 |
| Valine | 0.15 | -0.03 | 0.12 | 0.00 | 0.12 | 0.48 | 0.19 | 0.34 | 0.91 | 0.53 |
| Arginine | -0.18 | -0.27 | -0.09 | -0.11 | -0.05 | 0.24 | 0.22 | 0.15 | 0.20 | 0.36 |
| Citrulline | 0.24 | 0.12 | 0.10 | 0.12 | 0.04 | 0.12 | 0.11 | 0.19 | 0.31 | 0.20 |
| Glycine | -0.05 | -0.03 | -0.09 | -0.11 | 0.07 | 0.08 | 0.03 | 0.06 | -0.04 | 0.06 |
| Ornithine | 0.32 | 0.35 | 0.09 | 0.14 | 0.11 | 0.17 | 0.15 | 0.29 | 0.30 | 0.19 |
| Proline | 0.00 | -0.09 | -0.06 | -0.09 | 0.04 | 0.41 | 0.18 | 0.30 | 0.39 | 0.35 |
| Threonine | -0.10 | -0.11 | -0.09 | -0.13 | -0.04 | 0.25 | 0.34 | 0.25 | 0.34 | 0.51 |
| Serine | -0.01 | 0.04 | 0.01 | -0.01 | 0.21 | 0.06 | 0.29 | 0.11 | -0.02 | 0.09 |
| Histidine | 0.02 | -0.01 | 0.05 | -0.01 | 0.20 | 0.24 | 0.39 | 0.26 | 0.35 | 0.33 |
| Lysine | 0.12 | 0.12 | 0.07 | 0.07 | 0.18 | 0.10 | 0.32 | -0.02 | 0.22 | 0.19 |
| Tryptophan | 0.01 | -0.06 | -0.06 | -0.17 | 0.06 | 0.35 | 0.14 | 0.28 | 0.55 | 0.50 |
| Asparagine | 0.03 | 0.01 | -0.04 | -0.07 | 0.16 | 0.12 | 0.27 | 0.02 | 0.11 | 0.09 |
| Glutamine | 0.17 | 0.16 | 0.13 | 0.10 | 0.24 | 0.13 | 0.30 | -0.05 | 0.25 | 0.14 |
|  | Phenylalanine | Tyrosine | Valine | Arginine | Citrulline | Glycine | Ornithine | Proline | Threonine | Serine |
| C0 | 0.38 | 0.30 | 0.42 | 0.07 | 0.28 | -0.04 | 0.31 | 0.20 | 0.16 | 0.00 |
| C2 | 0.24 | 0.11 | 0.07 | -0.13 | 0.16 | -0.05 | 0.22 | -0.06 | -0.07 | -0.02 |
| C3 | 0.31 | 0.20 | 0.28 | 0.07 | 0.21 | -0.12 | 0.15 | 0.19 | 0.02 | -0.05 |
| C3DC | 0.27 | 0.08 | 0.11 | -0.17 | 0.15 | -0.13 | 0.16 | -0.02 | -0.14 | 0.14 |
| C4 | 0.19 | 0.15 | 0.12 | 0.02 | 0.07 | -0.01 | 0.08 | 0.05 | 0.03 | -0.04 |
| C4OH | 0.23 | 0.01 | -0.08 | -0.08 | 0.09 | -0.09 | 0.14 | -0.10 | -0.10 | 0.15 |
| C4DC | 0.15 | 0.16 | 0.11 | 0.06 | 0.18 | -0.05 | 0.17 | 0.12 | -0.01 | -0.20 |
| C5 | 0.39 | 0.26 | 0.31 | -0.09 | 0.21 | -0.11 | 0.27 | 0.25 | 0.06 | 0.11 |
| C5:1 | 0.22 | 0.12 | 0.10 | -0.07 | 0.08 | 0.01 | 0.13 | 0.02 | -0.02 | 0.40 |
| C5OH | 0.32 | 0.20 | 0.18 | -0.04 | 0.20 | 0.05 | 0.22 | 0.19 | 0.02 | 0.27 |
| C5DC | 0.33 | 0.15 | 0.22 | -0.14 | 0.31 | -0.13 | 0.26 | 0.16 | 0.06 | 0.06 |
| C6 | 0.17 | 0.11 | 0.07 | -0.06 | 0.04 | 0.05 | 0.12 | 0.23 | 0.01 | 0.15 |
| C8 | 0.18 | 0.14 | 0.11 | -0.08 | 0.06 | -0.01 | 0.18 | 0.22 | 0.02 | 0.00 |
| C8:1 | 0.16 | 0.07 | 0.08 | 0.01 | 0.14 | -0.03 | 0.19 | 0.07 | 0.02 | -0.03 |
| C10 | 0.17 | 0.11 | 0.10 | -0.10 | 0.07 | -0.03 | 0.18 | 0.19 | 0.00 | -0.05 |
| C10:1 | 0.18 | 0.14 | 0.11 | -0.05 | 0.08 | -0.02 | 0.18 | 0.21 | 0.07 | 0.00 |
| C12 | 0.20 | 0.09 | 0.10 | -0.17 | 0.17 | -0.08 | 0.23 | 0.08 | -0.08 | -0.07 |
| C14 | 0.20 | 0.03 | 0.04 | -0.18 | 0.17 | -0.09 | 0.21 | 0.02 | -0.14 | -0.07 |
| C14:1 | 0.16 | 0.04 | -0.01 | -0.17 | 0.13 | -0.08 | 0.21 | 0.05 | -0.11 | 0.00 |
| C14:2 | 0.19 | 0.09 | 0.07 | -0.10 | 0.18 | -0.03 | 0.23 | 0.13 | 0.02 | 0.00 |
| C14OH | 0.16 | 0.00 | 0.02 | -0.10 | 0.20 | -0.14 | 0.13 | -0.11 | -0.15 | -0.20 |
| C16 | 0.27 | 0.10 | 0.12 | -0.20 | 0.13 | -0.12 | 0.29 | 0.00 | -0.17 | 0.00 |
| C16OH | 0.27 | 0.07 | 0.10 | -0.14 | 0.19 | -0.14 | 0.20 | -0.03 | -0.14 | 0.08 |
| C16:1OH | 0.20 | 0.01 | -0.02 | -0.22 | 0.15 | -0.06 | 0.26 | -0.07 | -0.16 | 0.05 |
| C16:1 | 0.17 | 0.04 | -0.08 | -0.15 | 0.05 | -0.03 | 0.16 | 0.02 | -0.15 | 0.06 |
| C18 | 0.14 | 0.01 | 0.15 | -0.18 | 0.24 | -0.05 | 0.32 | 0.00 | -0.10 | -0.01 |
| C18:1 | 0.12 | -0.02 | -0.03 | -0.27 | 0.12 | -0.03 | 0.35 | -0.09 | -0.11 | 0.04 |
| C18OH | 0.20 | 0.06 | 0.12 | -0.09 | 0.10 | -0.09 | 0.09 | -0.06 | -0.09 | 0.01 |
| C18:1OH | 0.18 | 0.01 | 0.00 | -0.11 | 0.12 | -0.11 | 0.14 | -0.09 | -0.13 | -0.01 |
| C18:2OH | 0.15 | 0.02 | 0.12 | -0.05 | 0.04 | 0.07 | 0.11 | 0.04 | -0.04 | 0.21 |
| Alanine | 0.35 | 0.47 | 0.48 | 0.24 | 0.12 | 0.08 | 0.17 | 0.41 | 0.25 | 0.06 |
| Aspartic Acid | 0.33 | 0.25 | 0.19 | 0.22 | 0.11 | 0.03 | 0.15 | 0.18 | 0.34 | 0.29 |
| Glutamic Acid | 0.33 | 0.35 | 0.34 | 0.15 | 0.19 | 0.06 | 0.29 | 0.30 | 0.25 | 0.11 |
| Leucine | 0.61 | 0.58 | 0.91 | 0.20 | 0.31 | -0.04 | 0.30 | 0.39 | 0.34 | -0.02 |
| Methionine | 0.59 | 0.63 | 0.53 | 0.36 | 0.20 | 0.06 | 0.19 | 0.35 | 0.51 | 0.09 |
| Phenylalanine | 1.00 | 0.57 | 0.53 | 0.25 | 0.25 | -0.04 | 0.26 | 0.28 | 0.25 | 0.09 |
| Tyrosine | 0.57 | 1.00 | 0.60 | 0.18 | 0.19 | -0.03 | 0.24 | 0.38 | 0.38 | 0.02 |
| Valine | 0.53 | 0.60 | 1.00 | 0.17 | 0.27 | -0.08 | 0.29 | 0.36 | 0.35 | -0.10 |
| Arginine | 0.25 | 0.18 | 0.17 | 1.00 | 0.24 | -0.02 | -0.19 | 0.22 | 0.37 | 0.13 |
| Citrulline | 0.25 | 0.19 | 0.27 | 0.24 | 1.00 | 0.11 | 0.43 | 0.31 | 0.27 | 0.04 |
| Glycine | -0.04 | -0.03 | -0.08 | -0.02 | 0.11 | 1.00 | 0.11 | 0.07 | 0.15 | 0.51 |
| Ornithine | 0.26 | 0.24 | 0.29 | -0.19 | 0.43 | 0.11 | 1.00 | 0.27 | 0.24 | 0.13 |
| Proline | 0.28 | 0.38 | 0.36 | 0.22 | 0.31 | 0.07 | 0.27 | 1.00 | 0.37 | 0.06 |
| Threonine | 0.25 | 0.38 | 0.35 | 0.37 | 0.27 | 0.15 | 0.24 | 0.37 | 1.00 | 0.30 |
| Serine | 0.09 | 0.02 | -0.10 | 0.13 | 0.04 | 0.51 | 0.13 | 0.06 | 0.30 | 1.00 |
| Histidine | 0.36 | 0.33 | 0.29 | 0.16 | 0.08 | 0.00 | 0.08 | 0.14 | 0.31 | 0.33 |
| Lysine | 0.27 | 0.20 | 0.17 | 0.17 | 0.11 | 0.08 | 0.11 | 0.14 | 0.24 | 0.35 |
| Tryptophan | 0.41 | 0.57 | 0.55 | 0.10 | 0.09 | 0.03 | 0.21 | 0.23 | 0.27 | 0.16 |
| Asparagine | 0.12 | 0.11 | 0.07 | 0.09 | 0.09 | 0.19 | 0.06 | 0.13 | 0.18 | 0.37 |
| Glutamine | 0.26 | 0.18 | 0.21 | 0.18 | 0.16 | 0.05 | 0.16 | 0.15 | 0.25 | 0.38 |
|  | Histidine | Lysine | Tryptophan | Asparagine | Glutamine |  |  |  |  |  |
| C0 | 0.21 | 0.18 | 0.30 | 0.07 | 0.25 |  |  |  |  |  |
| C2 | 0.05 | 0.00 | 0.02 | -0.05 | -0.02 |  |  |  |  |  |
| C3 | 0.17 | 0.13 | 0.21 | 0.01 | 0.15 |  |  |  |  |  |
| C3DC | 0.15 | 0.17 | 0.11 | 0.10 | 0.22 |  |  |  |  |  |
| C4 | 0.02 | 0.02 | 0.02 | -0.02 | 0.02 |  |  |  |  |  |
| C4OH | 0.06 | 0.14 | -0.10 | 0.02 | 0.16 |  |  |  |  |  |
| C4DC | -0.01 | 0.00 | 0.09 | 0.02 | -0.06 |  |  |  |  |  |
| C5 | 0.24 | 0.21 | 0.26 | 0.07 | 0.23 |  |  |  |  |  |
| C5:1 | 0.31 | 0.25 | 0.23 | 0.17 | 0.31 |  |  |  |  |  |
| C5OH | 0.26 | 0.27 | 0.20 | 0.16 | 0.31 |  |  |  |  |  |
| C5DC | 0.18 | 0.16 | 0.11 | 0.11 | 0.21 |  |  |  |  |  |
| C6 | 0.11 | 0.18 | 0.03 | 0.13 | 0.16 |  |  |  |  |  |
| C8 | 0.08 | 0.13 | 0.03 | 0.08 | 0.07 |  |  |  |  |  |
| C8:1 | 0.02 | 0.05 | -0.05 | 0.00 | 0.05 |  |  |  |  |  |
| C10 | 0.02 | 0.08 | 0.00 | 0.04 | 0.03 |  |  |  |  |  |
| C10:1 | 0.08 | 0.16 | 0.02 | 0.10 | 0.10 |  |  |  |  |  |
| C12 | 0.02 | 0.06 | 0.01 | 0.01 | 0.06 |  |  |  |  |  |
| C14 | -0.01 | 0.09 | -0.06 | -0.01 | 0.09 |  |  |  |  |  |
| C14:1 | -0.01 | 0.09 | -0.08 | 0.01 | 0.09 |  |  |  |  |  |
| C14:2 | 0.03 | 0.08 | -0.04 | 0.04 | 0.08 |  |  |  |  |  |
| C14OH | -0.12 | -0.04 | -0.14 | -0.11 | -0.03 |  |  |  |  |  |
| C16 | 0.09 | 0.17 | 0.10 | 0.03 | 0.20 |  |  |  |  |  |
| C16OH | 0.05 | 0.14 | 0.01 | 0.01 | 0.19 |  |  |  |  |  |
| C16:1OH | 0.06 | 0.11 | -0.02 | -0.02 | 0.15 |  |  |  |  |  |
| C16:1 | 0.01 | 0.13 | -0.12 | 0.03 | 0.14 |  |  |  |  |  |
| C18 | 0.02 | 0.12 | 0.01 | 0.03 | 0.17 |  |  |  |  |  |
| C18:1 | -0.01 | 0.12 | -0.06 | 0.01 | 0.16 |  |  |  |  |  |
| C18OH | 0.05 | 0.07 | -0.06 | -0.04 | 0.13 |  |  |  |  |  |
| C18:1OH | -0.01 | 0.07 | -0.17 | -0.07 | 0.10 |  |  |  |  |  |
| C18:2OH | 0.20 | 0.18 | 0.06 | 0.16 | 0.24 |  |  |  |  |  |
| Alanine | 0.24 | 0.10 | 0.35 | 0.12 | 0.13 |  |  |  |  |  |
| Aspartic Acid | 0.39 | 0.32 | 0.14 | 0.27 | 0.30 |  |  |  |  |  |
| Glutamic Acid | 0.26 | -0.02 | 0.28 | 0.02 | -0.05 |  |  |  |  |  |
| Leucine | 0.35 | 0.22 | 0.55 | 0.11 | 0.25 |  |  |  |  |  |
| Methionine | 0.33 | 0.19 | 0.50 | 0.09 | 0.14 |  |  |  |  |  |
| Phenylalanine | 0.36 | 0.27 | 0.41 | 0.12 | 0.26 |  |  |  |  |  |
| Tyrosine | 0.33 | 0.20 | 0.57 | 0.11 | 0.18 |  |  |  |  |  |
| Valine | 0.29 | 0.17 | 0.55 | 0.07 | 0.21 |  |  |  |  |  |
| Arginine | 0.16 | 0.17 | 0.10 | 0.09 | 0.18 |  |  |  |  |  |
| Citrulline | 0.08 | 0.11 | 0.09 | 0.09 | 0.16 |  |  |  |  |  |
| Glycine | 0.00 | 0.08 | 0.03 | 0.19 | 0.05 |  |  |  |  |  |
| Ornithine | 0.08 | 0.11 | 0.21 | 0.06 | 0.16 |  |  |  |  |  |
| Proline | 0.14 | 0.14 | 0.23 | 0.13 | 0.15 |  |  |  |  |  |
| Threonine | 0.31 | 0.24 | 0.27 | 0.18 | 0.25 |  |  |  |  |  |
| Serine | 0.33 | 0.35 | 0.16 | 0.37 | 0.38 |  |  |  |  |  |
| Histidine | 1.00 | 0.57 | 0.39 | 0.36 | 0.47 |  |  |  |  |  |
| Lysine | 0.57 | 1.00 | 0.24 | 0.56 | 0.91 |  |  |  |  |  |
| Tryptophan | 0.39 | 0.24 | 1.00 | 0.27 | 0.22 |  |  |  |  |  |
| Asparagine | 0.36 | 0.56 | 0.27 | 1.00 | 0.51 |  |  |  |  |  |
| Glutamine | 0.47 | 0.91 | 0.22 | 0.51 | 1.00 |  |  |  |  |  |
