# Supplementary figures and images for: Metabolic signatures of insulin resistance in non-diabetic individuals
Source: BMC Endocr Disord. 2022 Aug 24;22:212. doi: 10.1186/s12902-022-01130-3 (PMC9404631; doi:10.1186/s12902-022-01130-3)

Additional file 2: Scree plot resulted from factor analysis


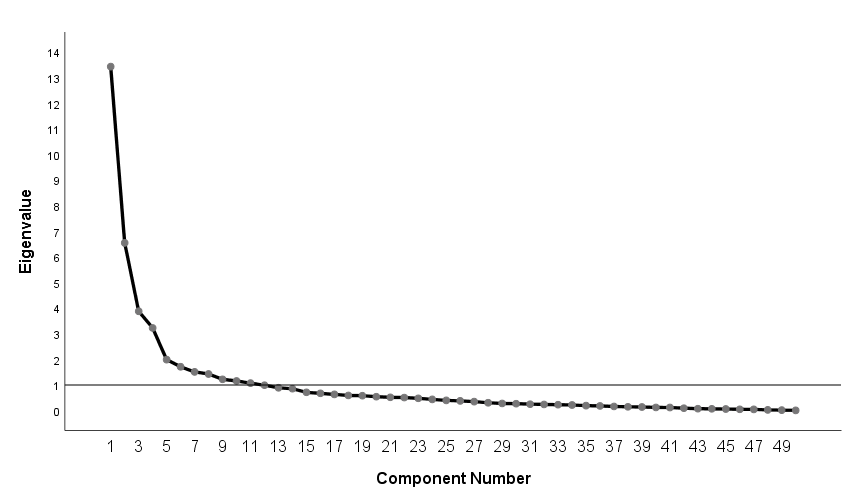

Supplement: Supplementary file 2 — Additional file 2. Scree plot resulted from factor analysis. [file 12902_2022_1130_MOESM2_ESM.docx]
