# Supplementary material for: Metabolic signatures of insulin resistance in non-diabetic individuals
Source: BMC Endocr Disord. 2022 Aug 24;22:212. doi: 10.1186/s12902-022-01130-3 (PMC9404631; doi:10.1186/s12902-022-01130-3)
Supplement: Supplementary file 3 — Additional file 3. Factor loadings of PCA considering Varimax rotation. [file 12902_2022_1130_MOESM3_ESM.docx]

Additional file 3: Factor loadings of PCA considering Varimax rotation

| **Component** | factor loading | **Component** | factor loading | **Component** | factor loading |
| --- | --- | --- | --- | --- | --- |
| **PC1** |  | **PC3** |  | **PC6** |  |
| C18:1 | 0.841 | Leucine | 0.814 | Citrulline | 0.796 |
| C16 | 0.832 | Tyrosine | 0.811 | Ornithine | 0.630 |
| C18:1OH | 0.831 | Valine | 0.805 | Proline | 0.426 |
| C16:1OH | 0.809 | Methionine | 0.783 | **PC7** |  |
| C18 | 0.801 | Tryptophan | 0.730 | Arginine | 0.795 |
| C16OH | 0.778 | Phenylalanine | 0.687 | Aspartic Acid | 0.363 |
| C14 | 0.747 | Alanine | 0.581 | **PC8** |  |
| C14OH | 0.738 | Glutamic Acid | 0.527 | Glycine | 0.807 |
| C18OH | 0.712 | Threonine | 0.439 | Serine | 0.695 |
| C2 | 0.690 | **PC4** |  | **PC9** |  |
| C16:1 | 0.656 | C5:1 | 0.840 | C18:2OH | 0.707 |
| C4OH | 0.611 | C5OH | 0.761 |  |  |
| C5DC | 0.449 | C5 | 0.664 |  |  |
| C0 | 0.341 | C3DC | 0.535 |  |  |
| **PC2** |  | C3 | 0.368 |  |  |
| C10 | 0.949 | **PC5** |  |  |  |
| C8 | 0.940 | Lysine | 0.918 |  |  |
| C10:1 | 0.908 | Glutamine | 0.890 |  |  |
| C6 | 0.778 | Asparagine | 0.704 |  |  |
| C14:2 | 0.755 | Histidine | 0.562 |  |  |
| C12 | 0.702 | Tryptophan | 0.730 |  |  |
| C14:1 | 0.667 | Phenylalanine | 0.687 |  |  |
| C4DC | 0.382 | Alanine | 0.581 |  |  |
| C8:1 | 0.326 | Glutamic Acid | 0.527 |  |  |
| C4 | 0.348 | Threonine | 0.439 |  |  |
| KMO measure=0.867; Bartlett's test result: p<0.001 | | | | | |
